# The “Dental Advertiser” and Its New Editor

**Published:** 1891-11

**Authors:** 


### THE "DENTAL ADVERTISER" AND ITS NEW EDITOR.

This quarterly journal announces a change in the editorial department, Thomas G. Lewis retiring, after a service of twenty-two years, and W. C. Barrett, M.D., D.D.S., taking his place.

The present number gives marked indications of his energetic work, and the editorials fairly bristle with his vigorous English. We congratulate this journal in securing the services of one who says exactly what he thinks, and means to be understood beyond any possibilities of doubt. Such men are rare, but are all the more valuable on this account.

It is a sign of the change coming, that the so-called trade journals are beginning to understand the fact that dentists are growing too intelligent to appreciate the cheap material ordinarily dealt to them under the name of dental literature. A high standard is demanded, and it is satisfactory to observe that the necessity for a change is being comprehended.

